# Qualitative study on the socio-cultural determinants of care of children orphaned by AIDS in the Ashanti and Eastern regions of Ghana

**DOI:** 10.1186/s12889-014-1332-7

**Published:** 2015-01-17

**Authors:** Lily Yarney, Chuks Mba, Emmanuel Asampong

**Affiliations:** School of Public Health, University of Ghana, Accra, Ghana; Association of African Universities, Accra, Ghana

**Keywords:** Children orphaned by AIDS, Social and cultural factors, Orphans care

## Abstract

**Background:**

Almost three decades after the discovery of HIV and AIDS in Ghana, the most obvious impact of the disease is the growing orphan crisis affecting most families and communities, especially in areas that the prevalence of HIV has exceeded the epidemic’s threshold of 5%. Studies have indicated that these orphans usually experience a wide range of problems which include education, nutrition, physical and sexual abuse, emotional and psychological distress, stigma and discrimination, among others The aim of the study was to examine the social, cultural, and traditional norms and practices that influence the care of children orphaned by AIDS in Ghana.

**Methods:**

The study employed focus group discussions, in-depth interviews and key informant interviews to generate information on the socio-cultural factors that impact orphan care in the Ashanti and Eastern regions of Ghana.

**Results:**

The findings indicated that the cultural practices that influence how well an AIDS orphan is taken care of by their caregivers include traditional rituals, ceremonies, and norms like funeral rites, marriage and naming ceremonies, festivals, inheritance, polygyny, and puberty rites. The social factors mentioned to affect orphan care significantly were: excessive alcohol drinking, and tobacco and drug use, dressing with fashion, night club attendance, market days, stigma and discrimination, among others.

**Conclusion:**

It is recommended that though some cultural and traditional practices are deeply rooted in communities and cannot be done away completely, orphan care policies on interventions should take into account these factors to mitigate their effects on the care of orphans.

## Background

Almost three decades after the discovery of HIV and AIDS in Ghana, the most obvious impact of the disease is the growing orphan crisis affecting most families and communities, especially in areas that the prevalence of HIV has exceeded the epidemic’s threshold of 5%. We define AIDS orphans as children under 18 years of age who have lost their mothers, fathers, or both parents to AIDS [1]. Studies have indicated that these orphans usually experience a wide range of problems which include education, nutrition, physical and sexual abuse, emotional and psychological distress, stigma and discrimination, among others [2-9]. A study also found that orphans under the patrilineal system of inheritance are better cared for than those under the matrilineal system [10]. These orphans tend to grow up in harsh environments and challenging circumstances. While ensuring the safety, wellbeing, and development of these orphans is challenging, the risks of a generation without adequate family support, education and opportunities are too great to be ignored.

Several studies have examined the situation and the plight of orphans across many locations in Africa, to include physical health, schooling, bereavement process, and psychological well-being [5,6,9,11-13], but lacking in these studies is the impact of socio-cultural practices and norms on orphan care, which are equally important if effective and meaningful interventions for orphans are to be achieved. Although community-based interventions are urgently needed as the most appropriate way of addressing the rising orphan problem, the complex local reality in which cultural factors, kinship ties and poverty are interwoven, needs to be taken into consideration if sustainable solutions are to be found [4]. Data of this nature are unfortunately limited in sub-Saharan Africa where problems of children orphaned by AIDS are most prevalent.

It is against this background that we undertook this study in two regions of Ghana, the Ashanti and Eastern, where the prevalence of HIV has been consistently high since 1986 when HIV and AIDS were first identified in the country. The objective was to examine the social, cultural, and traditional norms and practices that influence the care of children orphaned by AIDS in Ghana. These socio-cultural factors are essentially the totality of societal and population processes that shape and guide the people in their day-to-day dealings.

## Methods

The study was conducted in two purposively selected districts from the Eastern Region, and one metropolitan area and one district from the Ashanti Region, making a total of three study districts and one study metropolis. Within each selected district and metropolis, two communities were selected, yielding a total of eight study sites.

Children orphaned by AIDS and their caregivers were identified in the Ashanti Region through some non-governmental organizations (NGOs) working on HIV and AIDS and orphan care, in the study sites. In the Eastern Region, they were identified through staff of the Atua District Hospital, “Persons Living With HIV and AIDS” (PLWHA) and an NGO working with them.

In each of the two selected regions (within the districts and metropolis, and the selected communities within these areas), six groupings, made up of nine to twelve persons were selected purposively for Focus Group Discussions (FGDs). For the two selected districts in each of the study regions, two FGDs were conducted among adolescents – one for girls 12 to 17 years, and one for boys 12 to 17 years. Two FGDs were conducted among adults, one for women 18 years and above, the majority of whom were caregivers, and one for men 18 years and older. One FGD was conducted for orphans, and one for PLWHA. Thus, a total of 12 FGDs were conducted in the four selected districts in the two study regions. The purpose of the FGD was to collect spontaneous and general information on issues pertinent to orphans in the communities. In each of the study regions two traditional leaders were purposively selected for Key Informant Interviews (KIIs). Thus, four (4) KIIs were conducted at the regional level. Interviews with these persons were aimed at gathering information on the cultural norms and practices of their respective localities such as historical background, marriage, puberty rites, child rearing, inheritance issues, and their implications for orphan care. At the district and metropolitan level, Twenty (20) KIIs were held with district officials whose responsibilities had a bearing on HIV and AIDS and on orphan care, NGOs, FBOs, PLWHA, and Assembly men.

In-Depth interviews were also held at the community level with orphans aged between 10 and 17 years, and caregivers. In each of the study districts and metropolis, ten (10) in-depth interviews were conducted, with five (5) orphans and five (5) caregivers. Thus a total of forty (40) in-depth interviews were organised. The purpose of these interviews was to gather in-depth information from orphans and caregivers on how orphans are cared for, the issues that affect their care, their needs, feelings, perceptions and experiences.

The FGD and KII data collection instruments were pre-tested in purposively selected districts close to the study districts to determine their appropriateness for collecting the desired data. This exercise was used to test clarity, suitability as well as the logical flow of questions. The instruments were refined on the basis of issues that were raised and noted during the pre-testing exercise. The pre-test also helped to adapt the tools to the study objectives, and improved on the data collection techniques of the data collection team. Following the pre-test and subsequent revisions, the tools were rehearsed in Asante Twi language in the Ashanti Region and in Adangme language in the Eastern Region before they were used for the actual data collection.

The study was approved by the Ethical Review Committee of the Ghana Health Service (GHS) with identification number GHS-ERC: 13/3/08. Further, verbal and written consent were obtained from all illiterate and literate caregivers on behalf of all orphans. For those orphans who were less than 16 years, their caregivers were required to provide consent on their behalf to qualify them to partake in the study. All of them availed themselves willingly and without duress to participate in the study completely. Consent was also sought from all participants who were 16 years and above. The consent form which detailed the purpose of the study stated the fact that participation was voluntary and that participants could quit at any point in time during the process. Study procedures were thoroughly explained to all the participants and confidentiality was assured and maintained throughout the study. Consequently, all participants who agreed to be part of the study continued to the end of the study.

Responses from the Key Informant Interviews, in-depth interviews and Focus Group Discussions were first recorded using MP3 recorder and transcribed. The data were analysed on the basis of emerging themes and sub-themes in the context of the study framework. The findings on social practices and cultural norms and how they affect orphan care were grouped and presented in two main sections. The first section presents the cultural practices and norms whilst the second section presents the social factors reported to impact on orphan care in the two study regions. Findings were also presented in a bi-dimensional manner where possible, that is, social and cultural factors inherent in the study communities on the part of the caregiver that affect his or her ability to care for the orphan, and those on the part of the orphan that do not auger well for his or her care and wellbeing.

## Results

Table 1 shows the frequencies of the social and cultural factors that were mentioned by participants involved in FGDs, Key Informants Interviews, and in-depth interviews as affecting orphan care.

**Table 1:** **Socio-cultural factors impacting on orphan care**

**Factors affecting orphan care**	**Focus group discussions (12)**		**Key informants interviews (24)**		**In-depth interviews (40)**	
***Cultural factors***	***Freq.***	***%***	***Freq.***	***%***	***Freq.***	***%***
Traditional Ceremonies: Funeral Rites	12	100.0	23	95.8	36	90.0
Marriage Ceremonies	10	83.3	14	58.3	22	55.0
Out-dooring of Babies	10	83.3	14	58.3	20	50.0
Traditional Rituals: Festivals	8	66.7	16	66.7	15	37.5
Puberty Rites	10	83.3	18	75.0	25	62.5
Traditional Norms: Inheritance	12	100.0	19	79.2	20	50.0
Polygyny	7	58.3	12	50.0	13	35.0
***Social Factors***						
Alcohol and Hard Drugs	12	100.0	22	91.7	34	85.0
Social Eating and Drinking	8	66.7	14	58.3	21	52.5
Extravagant Living	6	50.0	13	54.2	23	57.5
Fashion	12	100.0	21	87.5	34	85.0
Sports and Games	10	83.3	16	66.7	15	37.5
Films/Video/Drama	7	58.3	15	62.5	18	45.0
Night Clubs/Discos	9	75.0	15	62.5	16	40.0
Traditional Dances	5	41.7	12	50.0	11	27.5
Market Days	7	58.3	14	58.3	24	60.0
Church Worship	6	50.0	13	54.2	14	35.0
Peer Influence	8	66.7	8	33.3	8	20.0
Stigma and Discrimination	10	83.3	17	70.8	28	70.0

### Cultural practices and norms and their impact on orphan care

Study participants in all the study districts and metropolis viewed traditional rites and cultural ceremonies as factors that affect orphan care in their communities. These include funerals; inheritance; marriage ceremonies, polygyny; naming ceremonies; puberty rites (especially among the Krobo); festivals and other traditional rituals.

#### Funeral rites and other traditional ceremonies

Traditional rites in Ghana usually cover the rights of passage of child-birth, puberty, marriage and death. These celebrations are considered important and memorable in the lives of people, and provide fulfilling moments to many families and communities [14]. However, funeral rites and marriage ceremonies (engagements and weddings) were reported to particularly affect orphan care in that a lot of money that could be useful in the care of orphans is diverted to organizing such traditional activities.

Funerals are celebrated in honour of the dead, thus, participants from both the Ashanti and Eastern regions view funerals as such, and therefore place a great deal of importance on the funeral ceremonies of family members and loved ones. Study participants from the Ashanti region believe that a person’s worth is unveiled at his funeral, hence, when an individual loses a relative, he is expected to perform befitting funeral rites, and if he is not able to do so, he is looked down upon by his community [15]. It is therefore not surprising that people attach much importance to funeral rites and would use money to buy clothes, shoes, bags, drinks, etc. and for public donations instead of using it for the provision of basic needs for children.

The Ashanti region participants identified about five phases of death, funeral rites and ceremonies that are costly and time consuming. These phases are imminent death, where the initial announcement of a person’s death involves purchasing of drinks and travelling long distances to inform distant relatives and significant others, printing of posters and the one-week celebration. Phase Two involves pre-burial and mourning when the place where the corpse will be laid in state is refurbished and decorated with expensive materials among other rituals. During this phase, community members are expected to make cash donations to the bereaved family. Study participants mentioned that if a community member does not make donations towards other people’s funerals, when he is bereaved, no one will make cash donations to support him, because ‘*woyε a nayεyε ma wo*’ (when you do it for others, they will also do it for you).

Phase Three is the interment which involves stuffing the coffin of the dead with expensive gifts from the widow, orphans, family and even friends. FGD participants indicated that some family members demand very expensive coffin and other items from the widow and her children to the extent that the widow is usually left with no money to immediately care for the needs of her children. Phase Four was described as the grand funeral which begins the day following the burial with widowhood rites and other preparations toward the grand funeral. Participants lamented that during these times, heavy sums of money are spent especially on the part of the widow and her children as well as the family of the deceased which may impoverish the widow for the rest of her life, thus:

*“She may never be able to educate her children afterwards, even what to eat may be a problem for the rest of their lives” (FGD, Women, Kumasi Metropolis).*

The last phase (Phase Five) is the periodic mourning where the dead is remembered during festive seasons like Easter, and *Adae* festivals where funerals are held for all the dead members of the community. Reports indicated that some people borrow money to perform funeral rites because they want to be respected socially, and they have to pay afterwards at times with interest, leaving them with little or no money to care for their families.

It was also reported in the Ashanti region that new clothes are purchased for almost every funeral, engagement/wedding or naming/out-dooring ceremonies. These festivities occur almost every day, therefore spending on new clothing, making donations, and other related expenses continue unabated. Such expenditures diminish the physical money (on the part of the caregiver) to be spent on caring for orphans and other family members.

*“It is traditionally acceptable to sometimes borrow money to organize extravagant funerals and pay for funeral donations, while the needs of children haven’t been provided for. Some people would buy new cloth for funerals, out-dooring, engagements and weddings, when their children do not have presentable school uniforms”* (FGD, Caregivers, Sekyere East District).

*“My mother* (*aunt*) *buys new cloth for every occasion*, *and I feel that is why she always complains of not having enough money to cater for the house and my schooling*” (Orphan, Sekyere East District).

It was also reported in both study regions that funerals are organized over long periods, and some caregivers hop from one funeral to another spending a lot of time with friends and other family members chatting, drinking and eating. Thus they spend very little time with their children and orphans leaving them on their own without adequate supervision. In addition, when more hands are needed in the preparation and organization of funeral rites and traditional ceremonies, some orphans are asked by their caregivers to assist instead of going to school.

In the Eastern region especially, the week-long funeral activities of drumming and dancing both day and night as reported by participants, finds many children including orphans, entertaining themselves. Some orphans place priority on such activities because that is their source of enjoyment and would even forego school during funerals and other traditional ceremonies.

“*Orphans leave school almost every week*-*end for funerals*, *and at times do not return to school*, *so we are forced to look for some other orphans to replace them in school*, *for us we have to meet our target*” (NGO Worker, Manya Krobo).

It was also mentioned that during such activities, adolescents and the youth learn to ingest alcoholic drinks, because they are usually served for free and this can be detrimental to their future if not controlled.

*Because alcohol is served free during funerals*, *everyone is served. Some of the children drink and become intoxicated. If this continues*, *they develop the habit of drinking alcohol*” (Assembly Man, Manya Krobo).

#### Traditional rituals

Two main traditional rituals reported by participants from both regions to affect orphan care are festivals, and puberty rites.

##### Festivals

In the Kumasi Metropolis, a lot of preparation is made towards the celebration of the *Adae Kese* festival which is held to climax the celebrations of some specific achievements and milestones of the Asante Kingdom. FGD participants and informants in the Sekyere East District mentioned both the *Adae Kese* and the *Papa* Festivals as events that seem to affect orphan care. In the Eastern region, the festivals mentioned are *Ngmayem*, *kloyomsikplemi*, and *Easter*. In all cases, participants explained that throughout the year people including the youth work, set aside money, and prepare to celebrate these festivals. New and expensive clothing are worn during festivals, lots of tobacco and alcohol are consumed, traditional dishes prepared and served, and lot of time spent drumming and dancing. In fact, to the majority of participants, the festival provides them the opportunity to re-unite with their loved ones and hence they must be very well prepared for that. As a result, money saved for festivals would not be used on things like paying school fees, feeding or paying for health services of orphans or other family members in need.

FGDs also revealed that some of the orphans and youth look forward to meeting old and new friends to enable them have fun. This may lead to teenage pregnancies, HIV and other sexually transmitted infections. When teenagers get pregnant, they hardly set eyes on the man responsible for the pregnancy through to their deliveries. Consequently, while some commit abortion and die, others die out of the stress of being single mothers, thus increasing the burden of orphans in the community:

“*A girl came to Manya from a nearby village to witness the festival*, *when she went back to the village*, *she realised she was pregnant but could not identify the man who impregnated her because she slept with more than one man. In fact*, *the Ngmayem Festival for the Manya area and the Kloyom Sikplemi for the Yilo area are presently doing more harm than ever to the youth especially orphans*” (NGO Worker, Manya Krobo District).

“*My niece became pregnant during the 1998 Ngmayem festival and could not tell who impregnated her*, *so the burden of care of the child has fallen on me*, *unfortunately I do not have enough money to take her to senior high school when she completes her basic education*” (FGD Men, Manya Krobo District).

##### Puberty rites

Puberty rites are performed for girls to mark the beginning of their maturity for marriage and sexual activity. Effects of puberty rites on orphan care were mostly mentioned in the Eastern region. It was explained that once puberty rites are performed, orphans are especially left to cater for themselves. Some of these girls go after men for their basic needs, stop schooling, and travel outside their usual places of residence and engage in commercial sex work. Some contract HIV and come back home to die. *Dipo*, the puberty rite of the Krobo of the Eastern region was reported as becoming a negative practice, because it is now exposing girls as young as seven years old to sexual activity with all its attendant problems.

“*Dipo is having negative effects on educating the girl child*, *because parents go for thire children from school for dipo. Consequently*, *these children absent themselves from school for more than three weeks*, *and some are never able to catch up with school work*” (NGO Worker, Yilo Krobo District).

“*Immediately after Dipo*, *my aunt* (*uncle*’*s wife*) *refused to give me anything I ask for*, *and I can tell from all indications that she feels I*’*m of age for a relationship with a man*, *so she does not see why I should continue bothering her and the husband financially*, *but I still go to school*” (Orphan,16 years, Yilo Krobo District).

“*I know of a nice girl in this community who vanished only a few months after Dipo had been performed for her*, *and now reports reaching us indicate that she is a sex worker in Abidjan*” (School Teacher, Manya Krobo District).

“*My sister*’*s daughter is of the same age as my daughter*, *so the family asked for Dipo to be performed for them*, *but I refused*, *so they started calling me names*, *I was forced to give my niece out for Dipo because I was the caregiver*, *and refused it for my daughter. Soon after*, *my niece travelled to Lome*, *and about two months ago*, *she came back very ill only to die later but my daughter is now in the university*” (Assemblyman, Manya Krobo District).

It was also indicated that some girls become rude and insolent after the rites have been performed for them, so their guardians or caregivers drive them away from their homes.

“*My elder brother asked my niece who is an orphan to leave his home because she was very disrespectful and rude to him after Dipo. Who knows*, *she may have a boyfriend who gives her money*” (FGD, women, Yilo Krobo District).

*Dipo* was also reported to negatively affect the schooling of girls because, as soon as it is performed, men start going after the girls, and most of them end up pregnant and therefore stop schooling.

Another traditional ritual that was mentioned in the Eastern region which impacts on orphan care is *Lapomi*. It involves the performance of the necessary rites by a man to claim children born out of wedlock since such children are regarded as belonging to the woman’s father. *Lapomi* can be very expensive and if this is done at the death of the mother of the children, the man is usually left with nothing to adequately cater for the orphans.

#### Traditional norms

The traditional norms reported to affect orphan care in the two study regions were Inheritance and Polygyny.

##### Inheritance

In the Eastern region, study participants reported that the patrilineal system of inheritance in itself is not a bad thing since children are supposed to inherit whatever property that belongs to their father. Ufortunately, what really happens is that, if parents especially the father dies whilst his children are young, the inheritor usually uses the inherited property for his own benefit and that of his own children. The inheritor sometimes totally neglects the widow and the orphans. Thus, property that is to be handed over to orphans when they grow up is taken up by the inheritor and keeps for his own children. The orphan therefore continues to suffer to a point of even becoming wayward and rebel when he grows up.

“*Patrilineal inheritance in itself is not a bad thing but most inheritors are greedy and use all the property for themselves and their own children*, *neglecting the orphans who should actually own the property had they not been children*” (FGD Men, Yilo Krobo District).

Patrilineal inheritance is indicated to be good for the orphan only when the deceased left behind some property that the caregiver can fall on in taking care of the orphan, otherwise it places financial difficulties on the caregiver.

“*Patrilineal inheritance is good*, *because if the dead father of the orphans has any property*, *it goes to his children*, *but if he leaves nothing behind as in the case of my brother and his wife*, *then the caregiver who might be the father*’*s relative as I am should struggle to take care of the orphans*” (Blind Caregiver, 70 years, Manya Krobo District).

Reports again indicated that if the deceased did not fully marry the woman with whom he had children, his property and wealth are transferred to his family and placed directly in the hands of his inheritor and his children. The orphans suffer neglect from the deceased’s family, and their care directly falls on the maternal family. If the woman also dies as is usually the case in HIV infection, care of orphans becomes the responsibility of the direct inheritor of the woman. In any case, the children may suffer depending on who the inheritor is:

“*Properties belonging to dead mothers of fatherless children by custom belong to the orphans*, *but are inherited by their maternal grandfather who may use the property for himself and his own children. Where there are farmlands he usually gives empty land which yields nothing to the orphans*” (Male Caregiver, Manya Krobo).

The natives of the Ashanti region on the other hand practice a matrilineal system of inheritance, and hold the belief that when a man dies, his children belong to their mother, as such, the deceased’s family neglect the orphans. Study findings however, indicated that tradition demands that the inheritor should also take care of the widow and orphans. On the contrary, most inheritors do not care for the orphans because of the perception that the orphans belong to their maternal family and that the rightful owners of the deceased’s property are his sisters’ children. The orphans according to the matrilineal system of inheritance are supposed to inherit from their maternal uncles, hence their responsibility to care for them. The people from the Ashanti region hold the perception that if any property of the deceased is transferred to the orphans, that property would be lost forever to the widow’s family. Consequently, the paternal family usually feels reluctant to assist the widow in taking care of orphans. This situation is captured in the following quotes:

“*Oheneba ne nea ne papa tease*, *egya bi wu a egya bi tease deε*, *yεde daadaa awisia*”(*The king*’*s child is the one whose father is alive. The saying that when one*’*s father dies*, *there is another one alive is only to woo the paternal orphan*) (FGD Men, Kumasi Metropolis).

“*I am taking care of two orphans*, *but the person who inherited their father does not care about them and would not assist me in anyway*” (Caregiver who is the maternal grandmother of orphans, Sekyere East District).

“*Our father*’*s family do not take care of us*, *because they say we belong to our mothers family*, *so the only person who cares for us is our mother*’*s sister*” (Orphan, Sekyere East District).

“*Sometimes*, *the one who inherits the dead father takes all the material things without taking care of the children*, *the maternal system of inheritance is really worrying*” (Caregiver, Sekyere East District).

“*I will say that matrilineal system of inheritance is very bad*, *because when my father died*, *his father* (*my grandfather*) *threw us out of the house that we were living in as a family*, *now my mother is very ill*, *and cannot take care of us. It is my aunt* (*mother*’*s sister*) *who is taking care of my mother and me. The remaining three of my siblings are living with people who are not family members. I recently heard that one of my paternal uncles wants to sell my father*’*s house*, *so I decided to inform another paternal uncle abroad. When I told him about it*, *his response was that*, *when the house is sold*, *he has a share in it because my father owed him some money. I was very disappointed and sad*” (Orphan, Kumasi Metropolis).

Study findings also indicated that under the matrilineal system of inheritance, if the man leaves a will or property for the wife and children, then the widow and orphans can have something to fall on, otherwise the paternal family takes everything from them and ends relationship with the widow and the orphans. Thus:

“*Even if the orphans greet them*, *they may not respond*” (FGD, Caregivers, Kumasi Metropolis).

##### Polygyny

Polygyny was indicated in the study regions as a cultural norm that affects the care of orphans. It was explained that some men who are caregivers of orphans marry more than one woman, limiting their resources to adequately cater for their children and orphans under their care. When this happens, it is the orphan that suffers most because he is the least of priorities. A man with more than one wife spends little time with each of his wives and children; hence children under his care do not have adequate supervision and engage in all kinds of unacceptable behaviours.

“*Gone are the days when men married many women so they can have a lot of children to assist on the farm. Presently every child is going to school*, *and the economic situation is difficult*, *so if you marry more than one wife and have many children with limited resources*, *taking care of them is difficult let alone the orphans under your care*. (Caregiver, Sekyere East District).

### Community social practices and their impact on orphan care

The social practices reported to impact on care of orphans include excessive alcohol, tobacco and hard drug use, rampant social eating and drinking, fashion, films, night clubs, games and sports, religious gatherings, and stigma and discrimination.

#### Alcohol and hard drugs

Alcohol use is common in many communities in Ghana, it is used for entertainment purposes, said to promote unity and togetherness, and for some, alcohol and drug use takes their minds off their problems or minimizes their hindrances. In all the twelve FGDs conducted in both study regions, and about 90% of informants of in-depth interviews indicated that alcohol, tobacco and other drug use impact heavily on orphan care. Actions and corresponding behaviours of alcoholics were identified and discussed among participants. Findings indicated that when people, especially men get drunk or are under the influence of hard drugs, their judgements and reasoning get impaired, become wild, and many women and children including orphans fall victim to some of their unintended actions, some even get raped and infected with HIV.

Such drugs are also addictive and once an individual is addicted, he or she will do all in his power to acquire it. Thus, when a caregiver is a drunkard or a drug addict, his first priority is to use his money for drinks or drugs. Drunkards usually lose control over orphans under their care, and do not show any good examples for the orphans to emulate. Some of these orphans may grow up and become alcoholics and drug addicts as well. Some women and orphan girls who abuse drugs and alcohol are usually taken advantage of by men.

It was mostly reported by orphans that alcoholic caregivers usually maltreat them because they see them as an economic burden. Some orphans reported that they are even afraid of going back home from school, because their drunkard caregivers may beat them, insult them, blame them unnecessarily, and even curse them sometimes.

“*It is very difficult living with an alcoholic father*, *because when he gets drunk*, *he doesn*’*t know where he puts his money*, *or whether he*’*s already spent it*, *he wakes up in the morning accusing you of stealing his money and starts beating you*” (FGD, Boys, Kumasi Metropolis).

#### Social eating, drinking and extravagant living

It was mentioned especially in the Ashanti region that people find it very entertaining spending time with friends in restaurants and bars, eating and drinking. People have developed such habits, and it is very difficult for them to stop when they marry and bear children or when they are supposed to be responsible for orphans. This lifestyle leaves them little money and time to take care of their dependants. Reports also indicated that for those who engage in such social activities, they prefer showing off to their drinking and eating counterparts by buying them food and drinks. Some women were reported to give money to their boyfriends for such purposes at the neglect of their dependants including orphans. Ostentatious spending favourite food and drinks from restaurants and bars were reported by some orphans as the reason why their caregivers cannot provide them with some of their needs.

“*My* ‘*mother*’ *likes sandwich and eggs*, *and she finds money to buy every day*, *but my school uniform is worn out and she never finds money to buy me a new one*” (Orphan, Kumasi Metropolis).

“*My uncle always comes home very late in the night because he spends a lot of time with his friends in the bar drinking and eating. He always comes home very satisfied and does not care whether we have eaten or not*, *he does not supervise our school work*” (Orphan, Kumasi Metropolis).

Study participants in both regions lamented that spending too much money and time on social activities render caregivers economically unable to provide for the needs of the orphans in their care. It also takes them away from home thus, neglecting some of the parental and supervisory roles. This makes some orphans vulnerable to many bad practices including pilfering and other social vices.

Placing priority on building houses was also mentioned as having effect on care of orphans. Some FGD participants indicated that some people place priority on acquiring physical properties than providing for the needs of their dependants. When this happens, orphans especially are affected and resort to other means of satisfying their needs.

“*My uncle has not been able to buy me books the whole of this year*, *but he is building a house that we go there to work at times*, *he always complains of not having enough money*” (Orphan, Yilo Krobo District).

#### Fashion

Fashion was mentioned as a factor that affects the care of children and orphans. Informants and FGD participants reported that women especially follow fashion and spend a lot of money on their hair, bleaching creams, new cloth and *kaba* styles, shoes, bags and the like. This puts strain on limited financial resources for the care of children and orphans. A number of orphans whose caregivers are women felt that, this is the main reason why they do not have adequate attention from their caregivers in the provision of their basic needs, especially food, clothing, education and health.

“*My mother buys every cloth in fashion and makes all the new styles available in town*, *but we do not have the freedom of eating what we yearn for like rice and chicken stew*, *because for her*, *that is luxury that she cannot afford quite frequently*, *so we only eat it at home during Christmas or when there is funeral in the family*” (FGD, Young Boys, Kumasi Metropolis).

#### Sports and games

Sports and games affect the care of children in the sense that some parents and guardians are addicted to them. Football was mostly mentioned, and it was explained that caregivers usually spend a lot of time with friends watching football in the evenings and on weekends, when they could spend that time with their children, and supervise their home work. In the rural areas, some caregivers will spend much time playing draught without doing any work to assist their dependants economically.

“*My uncle plays dame* (*draught*) *the whole day*, *and wouldn*’*t do anything apart from that*, *so when you ask him money for school*, *he will always refer you to his wife*, *because she is a petty trader*”(FGD, Young Boys, Sekyere East District).

Participants also explained that orphans and other children who play a lot of football and other games tend to neglect their school work, and do not do well in school. Some are influenced by friends into doing all kinds of bad things, including stealing to buy food from outside the home. On the other hand, some participants felt that football and other games help take the minds of children off engaging in social vices.

#### Films/video shows and drama

Film shows and drama are very entertaining and for caregivers who like watching them. They buy all the new films in town, or go to the theatre to pay and watch. These monies, FGD participants from both study regions explained, could be used to cater for the needs of children to enhance their quality of life.

Drama especially on television was mentioned as affecting the welfare of children in the sense that, what is watched is usually not monitored and these children learn bad behaviours from the films and drama they watch. Nigerian movies were particularly mentioned to portray violence. Children who watch television a lot also neglect their school work and usually do not do well in school, though it keeps them in the home most of the time.

#### Night clubs/discos and traditional dances

Dancing is viewed by most people as a social event that indicates happiness and unity. Some FGD participants and informants across both regions mentioned night clubs and traditional dancing as having effect on care of children and orphans. For caregivers who indulge in such activities, their finances are affected since such activities involve drinking as well, limiting their financial resources in taking care of the orphans in their care. In the Eastern region, it was reported that Fridays and Sundays are the ‘chilling’ days for most people.

Traditional dancing was especially reported among participants and informants in Sekyere East district of the Ashanti region where it was indicated that, during moonlit nights, the youth sing, play and dance. Some people dance and jump into the hands of their friends, and this can go on throughout the night. Children who engage themselves in these activities spend little time with their books and do not perform well in school. Orphans who have little or no supervision from their caregivers fall prey to such activities and their attendant problems.

#### Market days

Market days were particularly reported by informants from the Eastern region to affect orphan care because, most caregivers who are traders would ask the children under their care to assist them sell their goods. As a result, such children miss school at least once a week which affects their schooling. Seven (35%) orphans interviewed indicated that they go to school three or four times a week because they have to sell on market days to support themselves or their caregivers.

“*There is a very brilliant girl* (*orphan*) *in this school who misses school every Wednesday and Friday*, *because she has to assist her elder sister to sell tomatoes at the market in Agomanya*” (School Teacher, Manya Krobo District).

“*I go to school 3 times in a week because I sell toffees in the market on market days*: *this I do to assist my grandmother to take care of me*” (Orphan, Manya Krobo District).

“*My grandmother who I live with is 74 years*, *so I have to sell corn*-*dough on market days*, *thus I go to school three days in a week. If I don*’*t sell*, *there will be no money in the house for anything*” (Orphan, Manya Krobo District).

“*I go to the market on Wednesdays and Fridays to sell so I can have some money to buy food and other things for school since my sister cannot provide for me all the time*” (Orphan, 12 years, Manya Krobo District).

One Key Informant mentioned that some children intentionally refuse to go to school and opt to go to the market on market days only to meet their boyfriends and girlfriends.

“*Young girls are particularly vulnerable. On market days*, *men usually target them on the market*, *make friends with them*, *and have sex with them afterwards*” (NGO Worker, Manya Krobo District).

#### Church worship

About 50% of FGDs and some informants applauded church worship as having good influence on orphan care because churches teach their members to treat orphans well, and even ask them to make donations towards the care of orphans. It was however, reported that worshipping throughout the night during all night services takes caregivers from their homes at nights and the children under their care are left alone to cater for themselves. This gives the young boys and girls the opportunity to stay out of the home throughout the night, some join friends at funeral grounds, discos and night clubs where drinking and sexual activities go on.

Some young people who profess to be born again Christians gather at all-night prayers and worship and have sex afterwards. Caregivers would usually allow these children out the whole night thinking that they are out for prayers. Some reiterated that some pastors are involved in sexually exploiting women and young girls especially orphans and vulnerable children.

“*Some pastors use sweet words to convince the young girls to have sex with them*, *impregnate them and even infect them with HIV destroying their future*” (FGD, Men, Kumasi Metropolis).

#### Peer influence

Peers are found wherever teenagers go, school, funeral, night clubs, discos, and they constantly make friends. Participants explained that teenagers learn both bad and good behaviours from their peers. For those who find themselves in the company of good peers, they are influenced to become good children and they are liked by their caregivers, as such they also receive good care, but for those who have bad friends, they are influenced to become uncontrollable, disrespectful and ungrateful to their caregivers. Thus the caregivers feel reluctant to provide them with their needs.

“*It is very difficult to live with a child who trusts his friends more than you*, *even when you have good intentions for him*, *he does not appreciate it*, *and would point fingers at you as the bad one*, *this hurts too much*” (Caregiver, Sekyere East District).

Some caregivers are also said to be influenced by their peers, and this affects the care they give to the orphans they live with. Participants explained that some caregivers take instructions or suggestions from their friends as to how they should care for the orphans and this could be good or bad for the orphan.

#### Stigma and discrimination against orphans

Stigma and discrimination in the homes where orphans live were mentioned as affecting their wellbeing because where they exist, orphans are depressed. This affects their school work and their participation in other social activities. In the Ashanti region, it was explained that orphans are often discriminated against by their caregivers and their children when they go wrong. Some receive severe punishment which non-orphans do not for similar offences. Orphans are regarded as poor people, and do not deserve to use certain things in the home like a set of cutlery. Some even take different food from what the rest of the family is taking. For example, some orphans eat *waakye* or *koko* bought from the roadside every morning, whilst the rest of the family eats cooked food from the home.

Participants of FGDs and informants also mentioned that some orphans are discriminated against by their play mates, especially when they know that the parents died of AIDS. These orphans are accused of being witches, which is why they have lost their parents.

“*A child in our area is fond of telling an orphan they live with not to eat with a spoon because poor people don*’*t eat with cutlery*, *constantly reminding the orphan that she is poor and does not have to behave like other people whose parents can fend for them*”(FGD, Men, Kumasi Metro).

“*Someone insulted the child I am taking care of that he does not have parents*, *when the child did not know*, *and he came home crying bitterly*” (Maternal grandmother, Sekyere East District).

“*Some friends make fun of us when they have something we don*’*t have*, *some even go to the extent of telling us that we are the cause of our parents*’ *death*, *so they refuse to talk to us*, *and that can be very embarrassing*”(FGD, Orphan, Sekyere East District).

“*We are often beaten in the house*, *and insulted at the least provocation*” (FGD, Orphan, Sekyere East District).

In the Eastern Region, participants explained that orphans are discriminated against in the ways things are shared in the home, for instance, food, clothing, and education. They are discriminated against as to who should go to school amidst limited resources and who to continue to the highest educational level possible.

“*I feel so much unloved by my caregiver because she often insults and beats me*, *she also gives me little food*, *I*’*m much worried about this situation in the home*” (Orphan, Manya Krobo District).

“*In Krobo land*, *orphans are easily identified in the way they dress*, *schooling*, *feeding*, *etc. This is because most people including caregivers complain of limited financial and material resources*, *and most of what they have are used on their own children first before the orphan is considered*” (FGD, Adolescent Boys, Yilo Krobo District).

“*There is strong discrimination between orphans and non*-*orphans. We wear old and torn uniforms the whole year whiles our colleagues have new ones on. We are not given enough money for feeding at school. We do not have new clothing on occasions such as Christmas*, *Easter or even for Sunday church. We are teased by friends because we are poor and do not have parents. We are at times exposed to very bad things in the environment*, *and become confused and worried if we feel the need for these things*” (FGD, Orphans, Manya Krobo).

Some Caregivers however, claimed that the character of the orphan is also important in determining whether he or she would be discriminated against or not. According to them, some orphans are very bad and wicked children and it is difficult for the caregiver to consider spending money on them, especially when resources are limited.

“*Whether an orphan would be discriminated in the household or not also depends on his or her character and the availability of resources. At times there is very little money for the household*, *so the one that is obedient and respectful is considered first*” (Caregiver,Yilo Krobo District).

Eleven out of the 20 (55%) orphans interviewed felt that they are discriminated against physically by their caregivers and peers, whilst the remaining felt they are not discriminated against by anyone.

“*I don*’*t feel discriminated against*, *except that I feel I*’*m too poor*, *and that makes me uncomfortable in the midst of my peers*.” (Orphan Female 14, Manya Krobo District).

“*I don*’*t feel stigmatised or discriminated against by my aunt or peers. I feel that my aunt genuinely does not have the money to pay for my school expenses*.” (Orphan, Female, 12, Yilo Krobo District).

“*I am discriminated against by my brother*’*s wife*, *because when I need something*, *she will tell my brother not to give it to me because she feels I don*’*t need it*, *but her children are always given whatever they ask for*.”(Orphan, Male, 14, Manya Krobo District).

“*I am discriminated against by my aunt*, *because I*’*m not given what I need like school uniform. She often beats and insults me. I sleep on cloth on the floor whilst her children sleep on mattresses. Her children also insult me at times*.” (Orphan, Female, 13, Kumasi Metropolis).

“*I feel discriminated against by my aunt*, *because I am the only one in the house who wear old clothes during Christmas*, *she buys new ones for her children claiming that their father bought the clothes for them*, *yet I do all the hard work on the farm*.” (Orphan, Male, 12, Sekyere East District).

### Needs of asante and krobo caregivers

Money was indicated by all 20 (100%) caregivers involved in in-depth interviews as their primary need in carrying out their care-giving activities. Thirteen (65%) of them mentioned jobs that would provide them with regular monthly income as their need as well.

“*With money*, *I can provide for the needs of these children without any problem*, *but as at now*, *I must say it is very difficult for us*” (Caregiver, Sekyere East District).

“*I will say that we need more food*, *and clothing*, *but what is most pressing is money*, *since money can buy all that*” (Caregiver, Manya Krobo District).

“*I need a job to enable me earn some money*, *so that life can be a little bit easier for us”* (Caregiver, Kumasi Metropolis).

For those who are HIV positive, they requested in addition to money ‘Good Health’ to enable them take good care of the orphans in their custody.

“*My worst fear is that, if I’m no more, who will take of the children? I need good health, I need to be strong, so I can take care of the children, till they will be independent”* (PLWHA Caregiver, Manya Krobo District).

## Discussion

The results of the study have provided various explanations to the diverse socio-cultural determinants of orphan care in the study regions, thus, funeral rites and excessive alcohol or drug use and their effects on the care of orphans have been discussed further.

It is found from the study that the extravagance of funeral rites and ceremonies seems to affect the care of orphans in two ways. First, study participants indicated that much money is spent on the dead to the neglect of the needs of orphans and other vulnerable children. Second, the celebration of funerals is so elaborate that time for supervision and guidance of children is compromised, thus, providing opportunity for misdemeanour during the period when death is being celebrated. People from both the Ashanti and Eastern regions of Ghana organize funeral rites in the form of ritual observations as the hallmark of the personhood of the individuals both in this world and in the world of the ancestors and spirits. Hence, as noted from field notes the Asante, for instance go through about five phases of funeral rites and ceremonies that are carefully and routinely followed.

Study participants revealed that huge sums of money are usually spent, much of which are borrowed with high interest rates and this makes life difficult for orphans and their caregivers. In Asante funeral rites and ceremony, some deceased persons are even preserved in the mortuary for an extensive periods for the family to gather enough resources to enable them organize big funeral functions. These public functions provide the occasion for drunkenness, loss of working hours, and opportunities for immoral behaviours.

Some Christian leaders of the Kumasi Metropolis recognized the negative effects of how Asante funerals are organised and celebrated, and, in a pastoral letter signed in 1972 by the hierarchies of the Roman Catholic, Anglican, Methodist, AME Zion, and the Seventh-Day Adventist churches expressed grave concerns on the conduct of funeral rites in the Kumasi metropolis. The leaders noted that funerals had (i) assumed unnecessary proportions with high and indiscriminate use of alcoholic drinks which precipitated drunkenness, vices, quarrels, and fights; (ii) that neither the bereaved family nor sympathizers who donate money benefit from the expensive funerals. Without casting aspersions on funerals, the ministers were of the opinion that anything that is done in strict accordance with custom should not be tampered with provided that such a thing is not at variance with the Christian faith and good morality. They expressed further that they were aware of how much store the Ghanaian, and especially the individual who hails from the Ashanti region, lays by the funerals of his forbears, and indicated their appreciation and admiration for that. They therefore contended that the unbridled use of alcoholic drinks at funerals and the huge expenditures that have come to be inseparably associated with funerals these days, are not customary, and militate against traditional values.

Almost four decades after that letter was circulated in most communities in the Ashanti region, the problems of Asante funeral rites and ceremonies have gone beyond mere concerns over overwhelming expenditures and alcohol consumption. It has come to the notice of the Asantehene Otumfuo Osei Tutu II the negative impact of the manner in which Asante funerals are organized, and he has thus recently passed a ban on wake-keeping in Asante Kingdom. The effect of this is yet to be realised since the extravagant nature of Asante funerals still persists.

The situation is not too different from the Eastern region as indicated by participants. Modern Ghanaian funerals are therefore characterized by expensive preservations of corpses for prolonged periods, hiring of expensive hearses, elaborate customary rites, catered food, purchasing of fanciful funeral clothing, performances by musical groups, expensive custom-made caskets, and other related items and activities. The effect of this can only be detrimental to the future and wellbeing of orphans.

The effects of alcohol abuse were mentioned in all FGDs, and by most informants involved in the study. As indicated by participants of the study, apart from having economic implications on families and households where the head is an alcoholic or drug addict, excessive alcohol use by parents or caregivers also has certain social implications that can affect the future of children. Seven main aspects of family life that can be marred by alcohol misuse are identified as roles, routines, social life, communication, finances, conflicts and rituals [16].

It can be inferred from the study that problematic alcohol use by caregivers can affect the quality of their care-giving activities. This is because a caregiver who drinks may be inconsistent, emotionally unavailable, and unpredictable which can lead to passive neglect, and cruel care-giving. Children under such caregivers are not supported, nurtured or supervised [17]. Some children are even deprived of their childhood as they take on responsibilities that are beyond their age in the form of taking care of a sibling or a drunk parent or guardian, thus impairing their education and peer relationships, as they are also unable to flow with their friends as much as they should [18].

As indicated by some FGD participants in the Ashanti region, children under the care of parents or caregivers who have problem with alcohol or substance misuse go through emotional problems and this affects child–parent attachment throughout their life cycle [19]. Evidence also exists that children of problem drinking parents have higher levels of a range of problems than children of non-problem drinkers, and children of parents with other problems. The factors that could increase the likelihood of children being adversely affected include parental disharmony, violence, both parents drinking problematically, and the drinking taking place within the family home [20].

Some studies have also found links between domestic violence and alcohol consumption which is usually perpetrated by men against women [21]. A study on social work with families in which parents misuse drugs or alcohol found that alcohol misuse to be strongly associated with violence in the home [22]. The impact of domestic violence on children is often manifest in damage to family attachment, aggression or withdrawal, sleep problems, fear and a wish for safety [23]. By implication, a combination of a parent who has a problem with alcohol and who also suffers or perpetrates violence will exacerbate the harm and risk children face. Studies have also shown that some women resort to drinking alcohol to offset the impact of domestic violence on them [24-26].

Alcohol is noted to play a part in about 25% of known cases of child abuse [27]. Further, some research suggests that children are more likely to suffer physical abuse if the father is the drinker, and are more likely to suffer neglect if the mother is the drinker [17]. Thus orphans who live with alcoholic caregivers may be at risk from such violent and abusive behaviour, which may impact heavily on the orphans’ behaviour as they grow up.

The resultant impact of alcoholic parents or caregivers on children and for that matter orphans has been grouped into three, namely, (i) emotional problems; (ii) the school environment; and (iii) anti-social behaviour [16]. Emotional problems consist of a wide range of psychosomatic problems from asthma to bedwetting, negative attitudes to their parents and themselves, high levels of self-blame, withdrawal and depression. Problems associated with the school environment include learning difficulties, reading retardation, loss of concentration, generally poor school performance, aggression and truancy, whilst anti-social behaviour is manifested as increased risk of aggressive behaviour towards others, hyperactivity and other forms of conduct disorder. The researcher stated in addition that such children can have poor development of trust as found in this study (resulting from false accusations to abuse), problems with making and sustaining friendships, being a victim or witnessing conflict or violence, and verbal or physical aggression among others which do not auger well for the wellbeing of children and orphans.

### Limitation of study

The results of this study were specifically on orphans in the study area and may not be generalised to all children orphaned by AIDS. Also, the small numbers of children orphaned by AIDS and caregivers involved in the study place limitation on generalisation of findings to all children orphaned by AIDS in Ghana. Additionally, the plight of general orphans is unknown and this makes comparison of the plight of children orphaned by AIDS with that of general orphans difficult.

## Conclusion

The cultural practices found in the study to impact on the care of orphans included funerals, marriages, and naming ceremonies; traditional rituals such as puberty rites and festivals; and traditional norms like inheritance, and polygyny. The social factors, practices and activities found to influence the care of orphans were: stigma and discrimination, alcohol, tobacco/drugs, social eating/drinking, extravagant living, fashion, sports and games, films, video, and drama, night clubs and discos, traditional dance, market days, church worship, and peer influence. Most of these socio-cultural factors compete with orphan care for limited time and finance.

Socio-cultural patterns of life, such as funeral rites, puberty rites, festivals, inheritance, naming ceremonies, traditional dancing, among others, as found in the study are institutional and are passed on from generation to generation. As such, they are strongly cherished and allegiance to some of them is sometimes renewed regularly through the performance of rituals and rites. However, some of these patterns are found to negatively affect the care of orphans and children in general. Policies on interventions should therefore seek to make these practices safe and minimise their effects on orphan care.
